# Supplementary material for: Brain and psychological determinants of placebo pill response in chronic pain patients
Source: Nat Commun. 2018 Sep 12;9:3397. doi: 10.1038/s41467-018-05859-1 (PMC6135815; doi:10.1038/s41467-018-05859-1)
Supplement: Supplementary file 1 — Supplementary Information [file 41467_2018_5859_MOESM1_ESM.pdf]

Supplementary material for

Title: **Psychological and brain determinants of placebo pill response in chronic pain.**

Etienne Vachon-Presseau*[1], Sara E. Berger*[1], Taha B. Abdullah[1], Lejian Huang[1], Guillermo Cecchi[6] James W. Griffith[2], Thomas J. Schnitzer[3,4], A. Vania Apkarian[1,4]

[1]Department of Physiology,
[2]Department of Medical Social Sciences,
[3]Departments of Internal Medicine and Rheumatology,
[4]Department of Physical Medicine and Rehabilitation,
Northwestern University Feinberg School of Medicine, 710 N Lake Shore Drive, Room 1020, Chicago, IL 60611, USA

*EVP and SEB are co-first authors

12 Figures

7 Tables

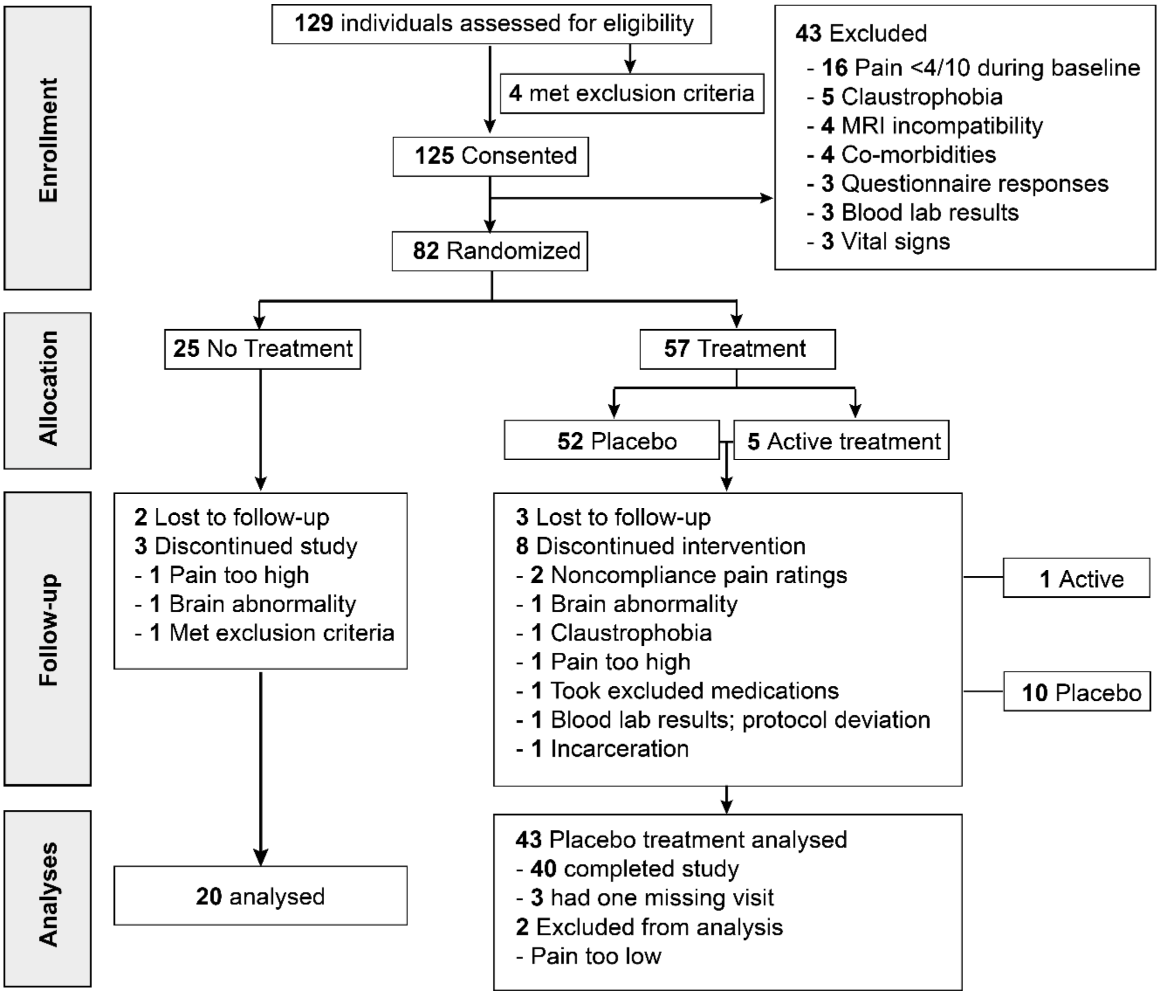

Supplementary Figure 1: CONSORT diagram. Disposition of all study participants from study entry to study completion, including those who failed at screening, those who discontinued, and those who successfully completed all study visits. Of the 129 people who were screened, 4 met exclusion criteria prior to consent; 125 individuals were consented and entered into the study. Of these, 43 screen-failed between visit 1 and visit 2 due to the reasons listed; the remaining 82 people were randomized into a no-treatment (n=25) or treatment (n=57) group according to a block design. Of the 25 individuals in the no-treatment group, n = 5 were either lost to follow-up or were discontinued post-randomization due to reasons listed, leaving a final n=20 that successfully completed all 6 visits of the study and were subsequently analyzed. Of the 57 treated individuals, 52 were allocated to placebo treatment and 5 were allocated to active treatment (Naproxen + Esomeprazole) in a double-blind fashion. After randomization, 10 placebo-treated individuals and 1 active-treated individual were either lost to follow-up or discontinued for reasons specified in the diagram. The remaining n=4 active treatment participants were not analyzed, since their only purpose was to aid in maintaining the double blind. The remaining n = 42 placebo participants successfully finished all study visits; however, 2 individuals were found to have baseline pain ratings not meeting eligibility criteria and were subsequently excluded, leaving n=40 placebo-treated individuals to complete the study. In addition to these participants, we added 3 people who had received all scans, but never made it to the final visit (i.e., they were only missing visit 6's interview and questionnaires); thus we ended up analyzing n=43 placebo participants.

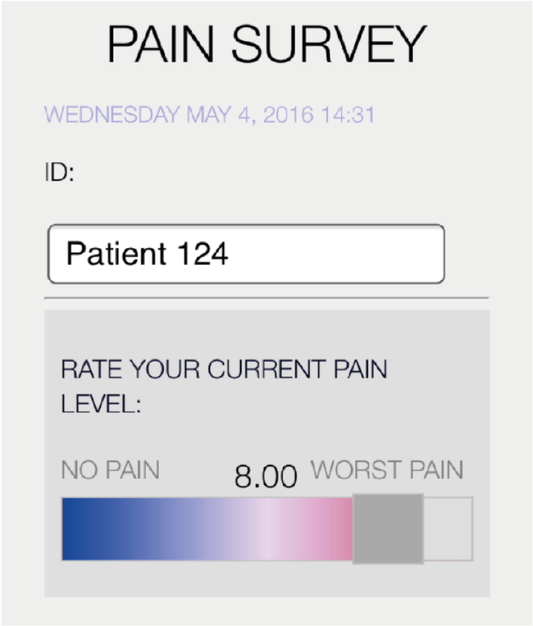

Supplementary Figure 2: Electronic Smartphone Application for collecting back pain intensity twice daily. Participants were asked to rate their pain twice a day for the duration of the study using a smart phone application (app) or computer. Pictured here is a screenshot of the app; participants entered their assigned ID and then rated their pain on a scale from 0 to 10, with 0 being no pain and 10 being worst pain. All ratings were sent to a secure server and stamped with the date and time completed. The app also had questions inquiring about rescue medication usage and treatment compliance (if applicable), and a comments section (not shown). Participants were paid $0.25 for each rating completed, up to $0.50/day.

	Age (years)	Female (%)	Pain duration (months)	Education (years)
PTx	46.1 (12.1)	14 (33)	52.8 (80.1)	12.3 (3.5)
NoTx	46.2 (13.2)	10 (50)	57.6 (61.2)	13.8 (3.7)
p_vals	0.98	0.18	0.81	0.15

Supplementary Table 1: Basic Demographics. There were no significant differences between treatment groups in age (unpaired t-test; $t_{(61)} = 0.024$, $p = 0.98$), gender (χ^2 (df:1)=1.76, p=0.185), duration of pain reported (unpaired t-test; $t_{(61)} = 0.237$, $p = 0.81$), or years of education (unpaired t-test; $t_{(61)} = 1.47$, $p = 0.15$). Table shows mean ± STD; all variables here were reported at visit 1.

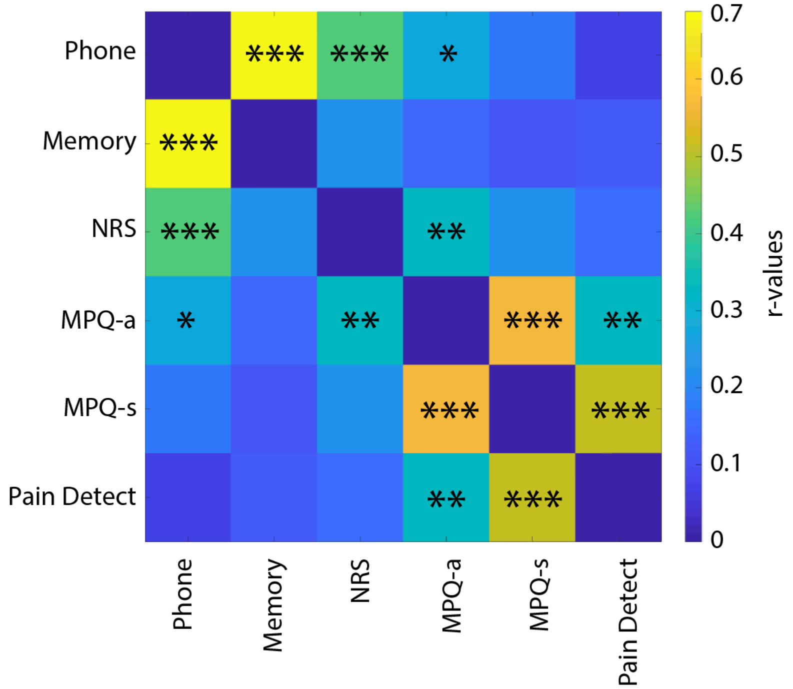

Supplementary Figure 3: Covariance matrix across pain assessments at baseline (Visit 2) across all patients. The intensity of pain was defined by the phone app ratings, memory of pain (a verbal report about the average pain experienced over the last 7 days), and a numerical rating scale (NRS collected in lab). The quality of pain referred to pain measurements from the MPQ affective (MPQ-a) and sensory (MPQ-s) scales, as well as the Pain Detect. The covariance was determined using Pearson correlations * $p < 0.05$; ** $p < 0.01$, *** $p<0.001$.

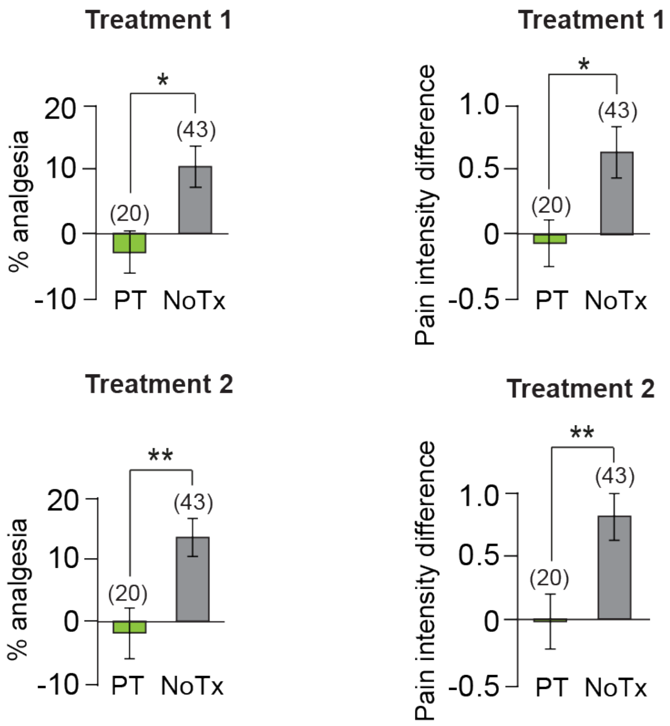

Supplementary Figure 4: Correspondence between %analgesia and the difference in the absolute scale scores between baseline and treatment periods. Compared to the NoTx group, PTx showed a 13.3% difference in average %analgesia from the phone app at treatment 1 (upper left, originally presented in Fig1c), which corresponded to an average of 0.71 units in absolute pain intensity (unpaired t-test $t_{(61)} = 2.24$; $p = 0.03$). Similarly, compared to the NoTx group, PTx showed a 15.6% difference in average %analgesia (lower left), which corresponded to an average of 0.84 units in absolute pain intensity (unpaired t-test $t_{(61)} = 2.65$; $p = 0.01$). We computed the effect size for the differences in absolute pain intensity scores, which corresponded to a Cohen's d' = 0.62 (95% CI: 0.33 to 0.90) for treatment 1 and Cohen's d' = 0.73 (95% CI: 0.45 to 1.01) for treatment 2. * $p<0.05$; ** $p<0.01$.

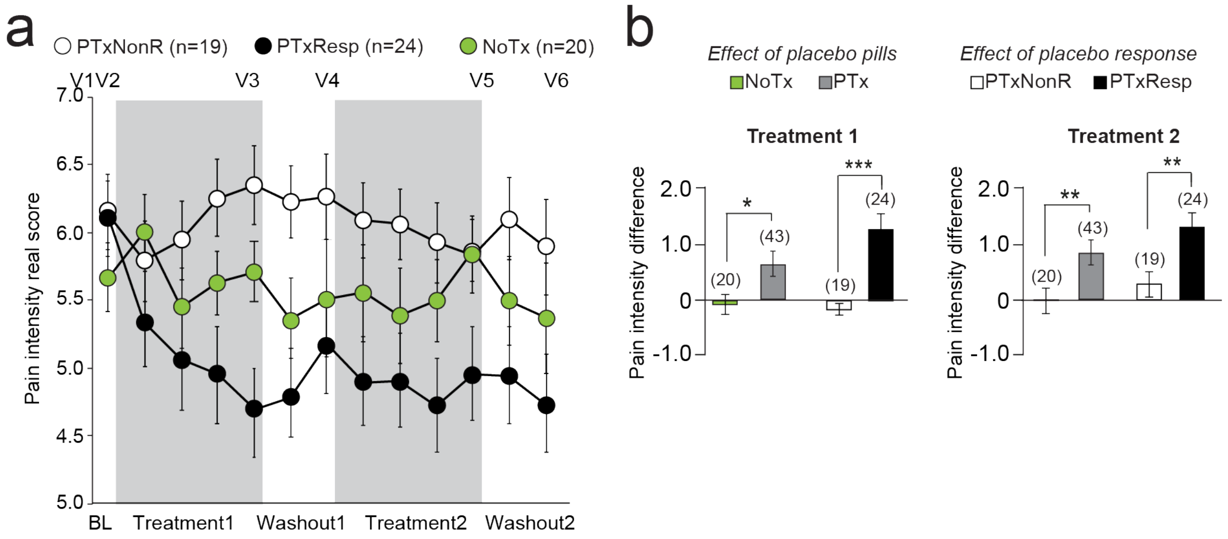

Supplementary Figure 5: The difference between baseline and treatment, in absolute scale, after stratification of PTx. a. The y-axis shows the pain analgesia in absolute scale (from the phone app) across the RCT. **b.** The PTx patients showed pain analgesia compared to the NoTx arm and PTxResp showed stronger analgesia than PTxNonR for both treatment periods (treatment 1: PTx Vs NoTx: unpaired t-test $t_{(61)} = 2.65$; $p = 0.01$; PTxResp Vs PTxNonR: unpaired t-test $t_{(41)} = 4.19$; $p < 0.001$); treatment 2: (unpaired t-test $t_{(61)} = 2.65$; $p = 0.01$); PTxResp Vs PTxNonR: unpaired t-test $t_{(41)} = 2.80$; $p = 0.008$). Compared to the NoTx arm, the PTxResp showed an average of 1.34 units in pain intensity difference in treatment 1 and 1.27 units in pain intensity difference in treatment 2. * $p<0.05$; ***$p<0.01$. Post hoc comparisons are Bonferroni corrected for 3 comparisons.

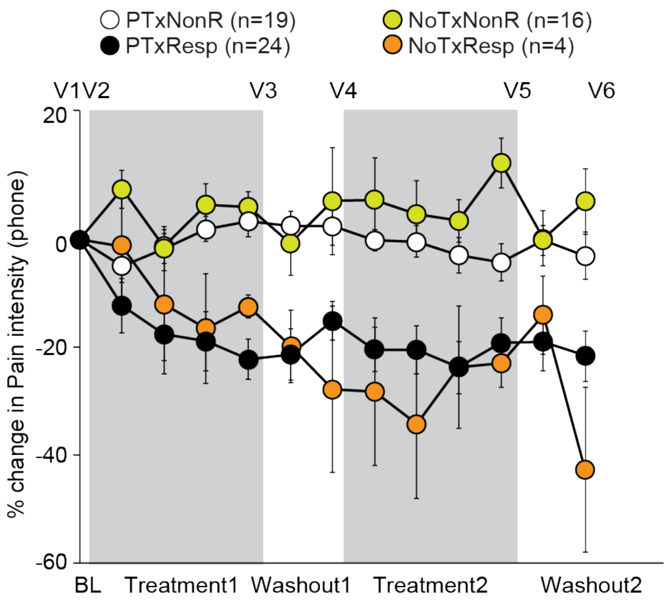

Supplementary Figure 6: The difference between baseline and treatment phone app ratings after stratification of PTx and NoTx. The pain trajectories are displayed for all 4 groups. A two-way repeated measure ANOVA show an interaction time*group: $F_{(14.79,276.13)} = 1.77$, $p = 0.04$. The frequency of responders was higher in the placebo pill treatment compared to no treatment: $\chi 2 = 7.09$, $p = 0.008$.

2a: Potential Confounds			
**Variable**	**Groups**	**Mean ± SEM**	**one-way ANOVA: F(2,60) = f-stat (p-value)**
**Pain app VAS Over Baseline**	PTxNonR<br>PTxResp<br>NoTx	6.23 ± 0.32<br>6.10 ± 0.20<br>5.68 ± 0.17	1.12<br>(0.33) n.s.
**Phone App Compliance**	PTxNonR<br>PTxResp<br>NoTx	81.14 ± 3.35<br>80.39 ± 2.67<br>75.77 ± 4.06	0.73<br>(0.49) n.s.
**Treatment Compliance**	PTxNonR<br>PTxResp<br>NoTx	90.7 ± 3.34<br>95.9 ± 3.84<br>n/a	**unpaired t-test: t-stat (p-value)**<br>0.73 (0.47) n.s.
**Rescue Medication Utilization**	PTxNonR<br>PTxResp<br>NoTx	7.5 ± 2.6<br>8.7 ± 2.5<br>3.5 ± 0.68	1.53<br>(0.23) n.s.

2b: Medicine Quantification		
**Groups**	**MQS Score ± SEM**	**one-way ANOVA: F(2,60) = f-stat (p-value)**
PTxNonR<br>PTxResp<br>NoTx	2.90 ± 0.76<br>3.22 ± 0.81<br>3.20 ± 0.78	0.05<br>(0.95) n.s.

2c: Medication Usage at Study Entry		
**Medications for Pain**	**# Patients (n = 63 total)**	**# Patients/Group**
**NSAIDs or acetaminophen**	24	7 PTxNonR<br>10 PTxResp<br>7 NoTx
**No treatment reported for pain**	32	10 PTxNonR<br>11 PTxResp<br>11 NoTx
**Combination of NSAIDs with other drugs**<br>• **Gaba-ergic anticonvulsants**<br>• **Muscle relaxers**<br>• **Opioids/narcotics**	6	2 PTxNonR<br>3 PTxResp<br>1 NoTx
**Gaba-ergic anticonvulsants**	1	1 NoTx

Supplementary Table 2: Placebo response is invariant to potential confounds. Study compliance and previous medication use are variables that could impact placebo response; we therefore tested these measures to verify that the placebo effect in our cohort could not be explained by these potential confounds. **2a.** There were no significant group differences in average pain rated using the electronic app over the 2-week baseline period, the percent compliance when using the electronic app to rate pain, compliance when taking the study agent, or the amount of rescue medication used. Compliance was considered across the duration of the study. One patient from the non-responder group failed to bring back his medications for either of the treatment periods, and thus study agent compliance could not be calculated for this patient. **2b.** The Medication Quantification Scale (MQS) was used to calculate a participant's pain medication usage at study

entry; an MQS score is based on the kinds of medications a patient is taking, the dosage of those medications, and the number of medications in total; there were no significant differences between groups in the total MQS composite score **2c**. The MQS was broken down into medications reported by participants to treat their pain, which included no treatment at all, NSAIDS or acetaminophen, and a combination of anti-inflammatories with other drugs, such as gaba-ergic medicines, muscle relaxers, and opiates/narcotics. There were no differences in the amount of patients per group using these medications (group X medication-category, $\chi^2 = 3.16$, $p = 0.79$). All participants were required to discontinue all medications used to treat their pain for the duration of the study beginning at visit 1. Unless otherwise stated, PTxNonR = 19; PTxResp = 24; and NoTx = 20.

Pain Outcomes by Treatment Session (% change in score)	Treatment 1	Treatment 2
	VAS (phone app)	VAS (phone app)
NRS	**r = 0.46, p = 0.003, n = 40**	**r = 0.55, p = 0.003, n = 39**
Pain Memory	**r = 0.72, p <0.001, n=40**	**r = 0.45, p = 0.004, n = 39**
MPQ-sensory	r = 0.06, p = 0.71, n = 42	r = - 0.06, p = 0.67, n = 41
MPQ-affective	r = 0.21, p = 0.23, n =34	r = - 0.16, p = 0.39, n = 31
PainDetect	r = 0.05, p = 0.75, n = 41	r = 0.13, p = 0.42, n = 39

Supplementary Table 3: Comparison of treatment outcomes with phone app ratings in PTx. All pain-related scores were first converted to percent change from baseline for both treatment sessions. Table shows Pearson correlations between the percent change using the smart phone app (from which we based our stratification of placebo responders/non-responders) with percent change in other pain outcomes. Pain questionnaires using numerical scales such as the verbal pain memory report and the Numeric Rating Scale (NRS) significantly correlated with the phone app. Of the remaining pain outcomes, only the Neuropathic Pain Scale (NPS) showed modest correlation with the VAS from the phone app, which was for treatment 2 only. Correlation coefficients, p-values, and corresponding number of patients are provided.

Category	Questionnaire	Visits Administered	Description	References
**General**	MQS	1	*Medicine Quantification Scale*: used to score how much and what kinds of medications participants were using before or at the start of the study; if participants were taking concomitant pain medications, they were asked to stop these for the duration of the study	[1]
	PHI	1	*Personal Health Information*: collected a general medical history, a history pertaining specifically to back pain – including causes, previous treatments, or surgeries – and information about education, income, sleep, smoking, and alcohol usage.	n/a
	Demographics	1	*NIH Demographics Form:* Collected gender, race, and ethnicity	[2]
**Pain outcomes**	NRS	all	*Numeric Rating Scale*: provides an additional pain rating from 0 (no pain) to 100 (worst pain imaginable); standard method of collecting perceived pain level in a clinical setting	n/a
	MPQ-sf	all	*McGill Pain Questionnaire (short form)*: measures location, duration, intensity, and quality of pain	[3]
	NPS	all	*Neuropathic Pain Scale*: measures the neuropathic components of participant's pain	[4]
	pDT	all	*painDETECT*: provides information about location, duration, intensity, and quality of pain at different time scales	[5]
**Pain & emotions**	CPAQ	1	*Chronic Pain Acceptance Questionnaire*: measures the effort participants put into either actively controlling their pain (activity engagement subscore) or passively accepting their pain (pain willingness subscore)	[6, 7]
	CPCI-42*	all	*Chronic Pain Coping Inventory*: measures 3 kinds of maladapative coping strategies (guarding, resting, and asking for assistance) and 6 kinds of adaptive coping strategies (exercising or stretching, relaxation, task persistence, purposeful self-statements, pacing activity, and seeking social support) in response to chronic pain; the idea is that certain illness-focused, maladaptive behaviors are associated with more disability, where as some wellness-focused behaviors are linked with less disability or recovery.	[8, 9]
	PCS	1	*Pain Catastrophizing Scale*: assesses how much people worry about their pain and its possible causes (yields a total	[10]

			score and subscales of rumination, magnification, and helplessness)	
	PASS-20	all	*Pain Anxiety Symptoms Scale*: measures pain-related fear, avoidance, and anxiety	11
	PSQ	1	*Pain Sensitivity Questionnaire:* assesses participants' sensitivity to imagined painful and non-painful stimuli	12
**Study agent efficacy**	GIC	all	*Global Impression of Change*: to score the perceived change of pain from one visit to the next on a 5-point scale, including much better, better, no change, worse, and much worse.	n/a
	TSS	3,5	*Treatment Satisfaction Survey*: lab-developed, un-validated survey asking individuals to rate on an 11-point scale (-5 to +5) how satisfied or dissatisfied they are with the study treatment and to explain why	n/a
**Expectations and beliefs related to medications and health**	SETS	2,4	*Stanford Expectations of Treatment Scale*: assesses participants' positive and negative expectations towards the upcoming treatment, and their overall level of understanding of the treatments' purpose	13
	PSM	all*	*Perceived Sensitivity to Medication*: measures how sensitive people think they are to medication in general, which might affect if they respond to a placebo	14
	HCAMQ	6	*Holistic Complementary and Alternative Medicine Questionnaire*: assesses via two subscores participant beliefs about whether alternative and complementary medicinal techniques work and should be used; we were interested in seeing if these beliefs were correlated to placebo response	15
	MHLC – C	6	*Multidimensional Health Locus of Control (form C)*: Assesses what factors participants believe are responsible for, and in control of, their health (themselves, chance, or other people to various degrees)	16
**Mindfulness and Emotional Control**	MAIA	1	*Multidimensional Assessment of Interoceptive Awareness*: measures the extent to which someone is aware of his/her body and emotions and how well they can either focus or distract themselves from these sensations (8 subscales reflect various aspects of this awareness); additionally, scores on the MAIA have been shown to be lower in individuals with back pain	17, 18
	ERQ	1	*Emotional Regulation Questionnaire*: measures two kinds of strategies people use to control their positive and negative	19

			emotions (including a re-appraisal and a suppression subscore)	
	ACS	1	*Attentional Control Scale*: assesses the voluntary control of attention during a variety of situations	[20]
	eACS	1	*Emotional Attentional Control Scale*: assesses the voluntary control of attention during emotionally demanding situations, which could include pain	[21]
	FFMQ	1	*Five Facets of Mindfulness Questionnaire*: a combination of many well-known and validated questionnaires, this measures the five main components of mindfulness as a skill set, which may correspond to placebo propensity.	[22]
**Suggestibility**	MISS-sf	6	*Multidimensional Iowa Suggestibility (short form)*: measures the extent to which participants can be influenced by a variety of other external and internal factors	[23]
**Personality**	NEO-FFI	1	*NEO Five* Factor Inventory: measures participants' scores on personality dimensions (extraversion, agreeableness, conscientiousness, neuroticism, and openness); previous research has shown that personality plays a role in placebo response in healthy controls	[24, 25]
	LOT-R	1	*Life Orientation Test (Revised)*: measurement of dispositional optimism, which has been shown to influence placebo propensity in healthy individuals	[26]
	LAQ	1	*Loss Aversion Questionnaire*: measures how sensitive participants are to a wide variety of potential "losses" in their lives. Since we have already published results showing that chronic back pain patients are more gain sensitive (less loss averse[27]), we were interested in investigating whether this trait also affected propensity to respond to placebo	[28]
**Affective State**	BDI-Ia	1,3,5	*Beck Depression Inventory, Version 1a:* measures the extent to which a participant may be clinically depressed; a score of >=19 was an exclusion criterion for the study	[29]
	PANAS	all	*Positive and Negative Affect Schedule:* assesses the extent to which participants are feeling a list of positive and negative emotions on the day of the visit to try to quantify the current affective state	[30]

Supplementary Table 4: List of questionnaires administered to patients. 29 self-report measures (47 measures total if divided into respective subscales) were completed at designated

visits in the study. The name and abbreviation are provided for each of the questionnaires, along with the rationale for why each measure was included in our battery. Also included are the visit(s) at which each measure was administered. An asterisk marks a questionnaire that wasn't analyzed due to poor questionnaire compliance through either selection of extremes or skipping items (CPQI). Only questionnaires administered at V1 were used in the machine learning models.

Group	Positive V2	Negative V2	Positive V4	Negative V4	Delta Pos (V4-V2)	Delta Neg (V4-V2)
PTxResp (n=18 V2; n = 19 V4)	4.44 (0.96)	3.04 (1.62)	3.69 (1.58)	2.26 (1.43)	-0.65 (1.20)	-0.77(1.63)
PTxNonR (n = 14 V2; n = 16 V4)	4.19 (0.94)	3.07 (1.47)	4.37(1.49)	2.92 (1.45)	-0.06 (1.31)	-0.08 (0.81)
Two-sample t-test	t = -0.75; df(30); p=0.46	t = 0.06; df(30); p=0.95	t = 1.30; df(32); p=0.20	t = 1.34; df(32); p=0.19	t = 1.24; df(26); p=0.23	t = 1.34; df(26); p=0.19

Supplementary Table 5: There were no significant differences in positive or negative expectations or in the changes in expectations following treatment 1. There were a few subjects that did not complete the SETS questionnaire. The number of subjects are presented for each analysis.

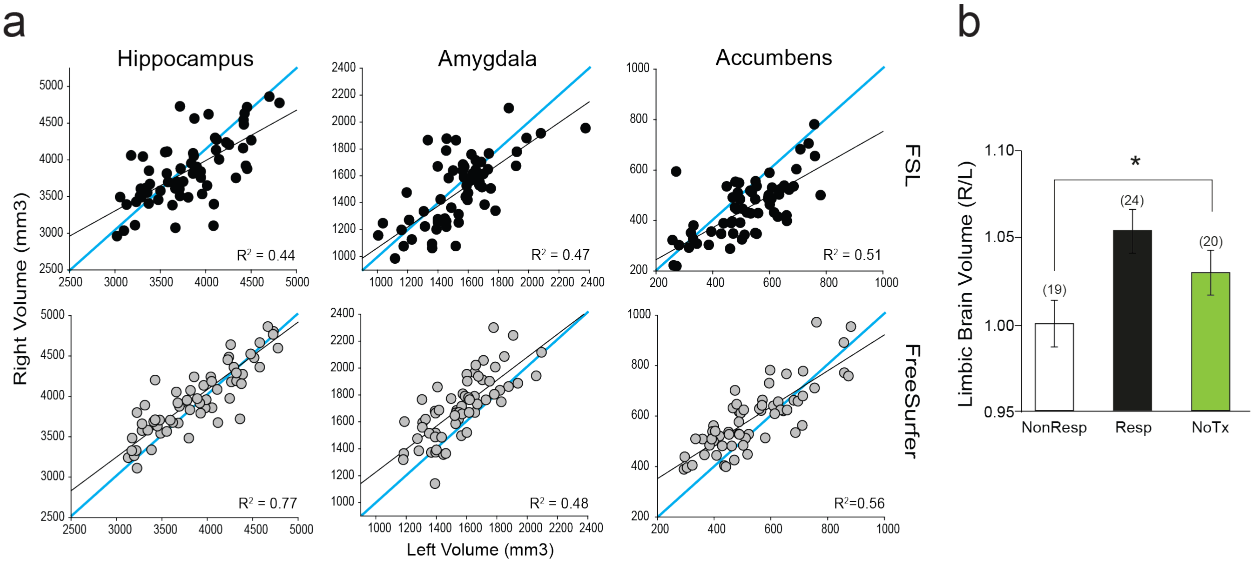

Supplementary Figure 7: Limbic volume asymmetry found using both FSL and Freesurfer. Subcortical volumes were segmented and extracted bilaterally for 3 regions of interest: hippocampus, amygdala, and nucleus accumbens; this was done twice using two different softwares – FSL and Freesurfer. **a.** For both FSL and Freesurfer, volumetric asymmetry between hemispheres was found for all regions. Scatterplots show Pearson correlations between right and left volumes of the hippocampus, amygdala, and nucleus accumbens for FSL (black, top) and Freesurfer (gray, bottom) for all patients. X-axis = left hemisphere; y-axis = right hemisphere. Blue lines represent a 1/1 ratio (identity line) where left and right volumes would be equal to each other; values above this line indicate more rightward asymmetry, whereas values below indicate more leftward asymmetry. **b.** The volumes from all 3 regions were summed for each hemisphere and represented as a ratio, with total right limbic volume in the numerator and total left limbic volume in the denominator. Freesurfer's segmentation replicated the significant differences between responders and non-responders initially observed using FSL ($F_{(3,56)} = 2.78$; $p = 0.049$; one-way ANCOVA between 3 groups with age, gender, and peripheral grey matter as covariates). Results from only the correct classification (classifier #3 for subcortical volume analysis) is shown for all plots. $^{*}p < 0.05$ after Bonferroni correction for 3 comparisons.

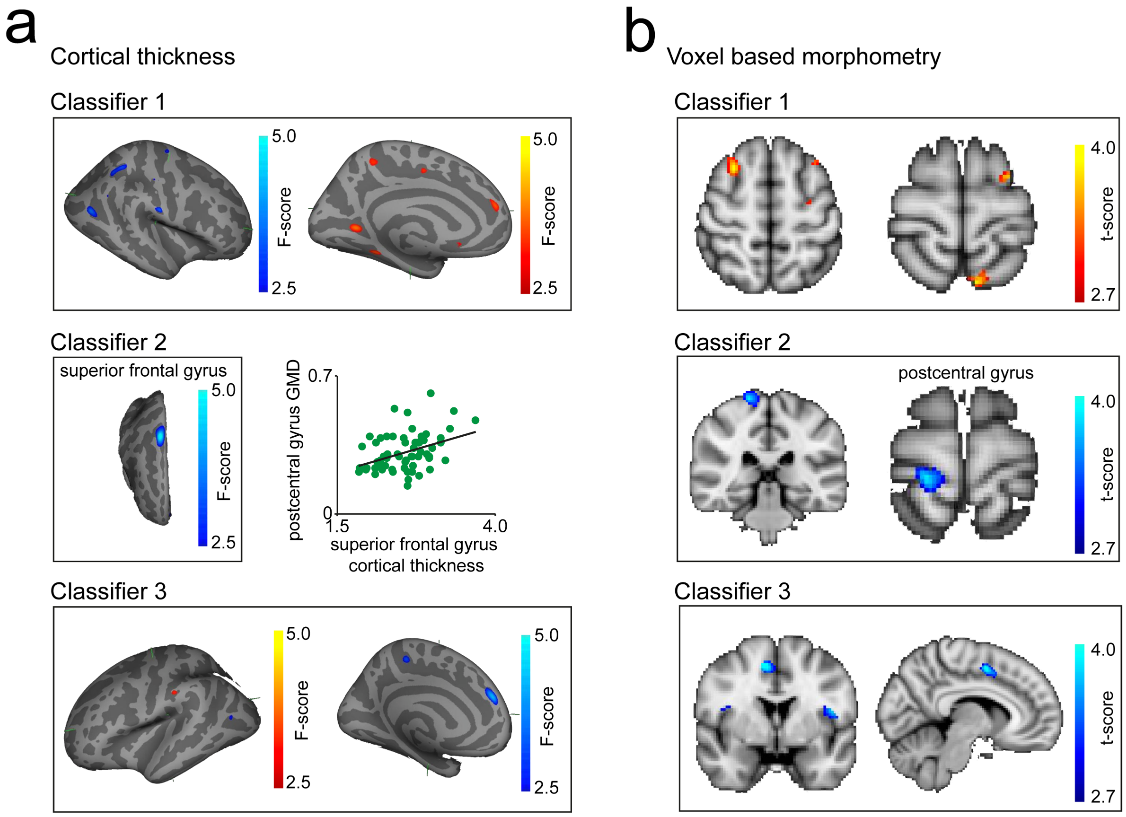

Supplementary Figure 8: Placebo non-responders showed higher grey matter density in the postcentral gyrus (Classifier 2). Cortical properties were examined with cortical thickness and grey matter density (GMD). **a.** All T1-weighted images were first brain extracted and then segmented into grey matter, white matter, or cerebrospinal fluid using FSL software. For the cortical thickness, only classifier 2 yielded significant random-field-theory-based significant clusters. For the GMD, all three classifier codes generated results that did not survive Threshold Free Cluster Enhancement (TFCE). Clusters from Freesurfer and GMD were both located within nodes functionally defined as the sensorimotor community (in **fig. S4**). They correlated together (r = 0.4; p = 0.001), suggesting a common mechanism.

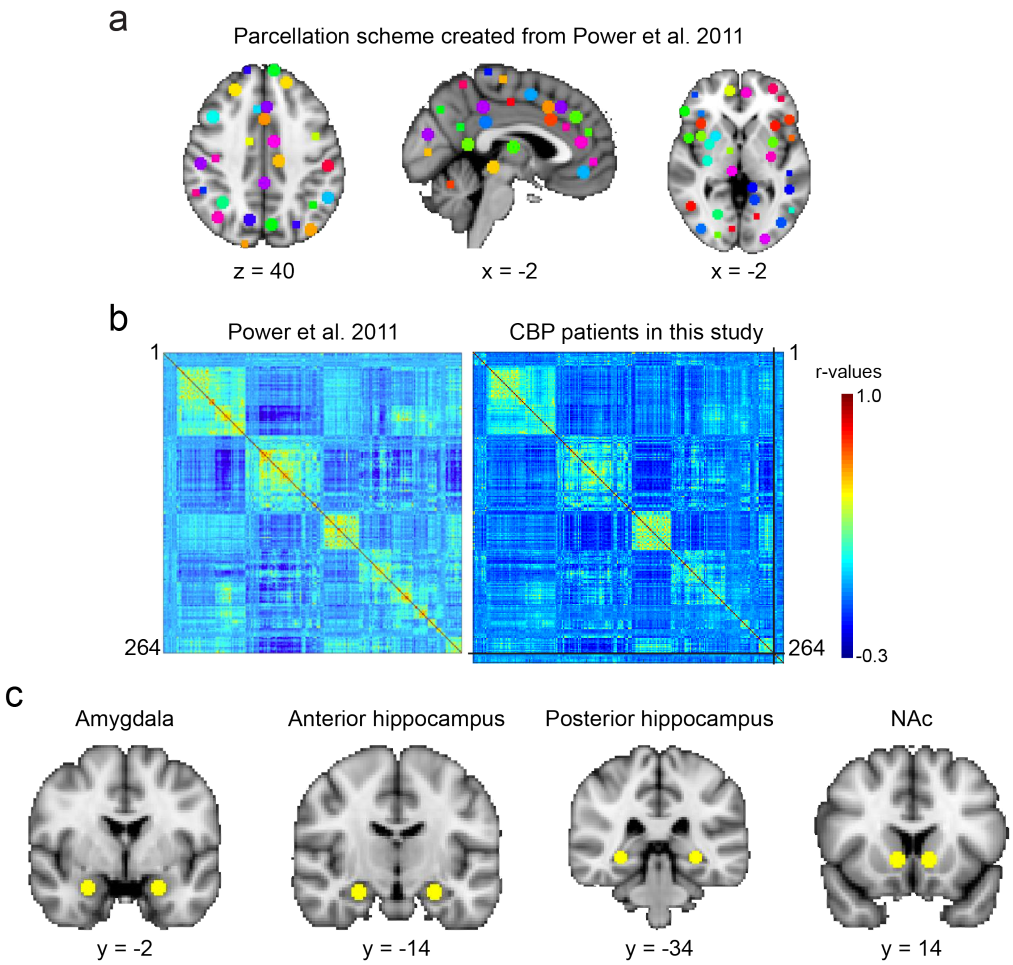

Supplementary Figure 9: Parcellation of the brain into 272 ROIs. a. In this study, we used the parcellation scheme from Power et al 2011[31]. A parcellation was constructed from functional brain networks of 5-mm radius ROIs located in 264 regions, representing the putative functional areas of the brain. These regions were identified from a meta-analysis on task-based fMRI and complemented by using the center of gravity of cortical patches constructed from resting state functional connectivity. **b.** The similarity between the averaged connectivity matrices of 106 healthy subjects from Power's study with the averaged connectivity matrices from our 63 CBP patients can be visually appreciated. The ROIs extending below the black line (ROIs 265-272) represent the ROIs that were manually added in subcortical regions of interest displayed in **c**. **c.** These limbic ROIs, representing 5-mm radius parcels, were added in amygdala, bilateral anterior hippocampus, bilateral posterior hippocampus, and bilateral nucleus accumbens (NAc).

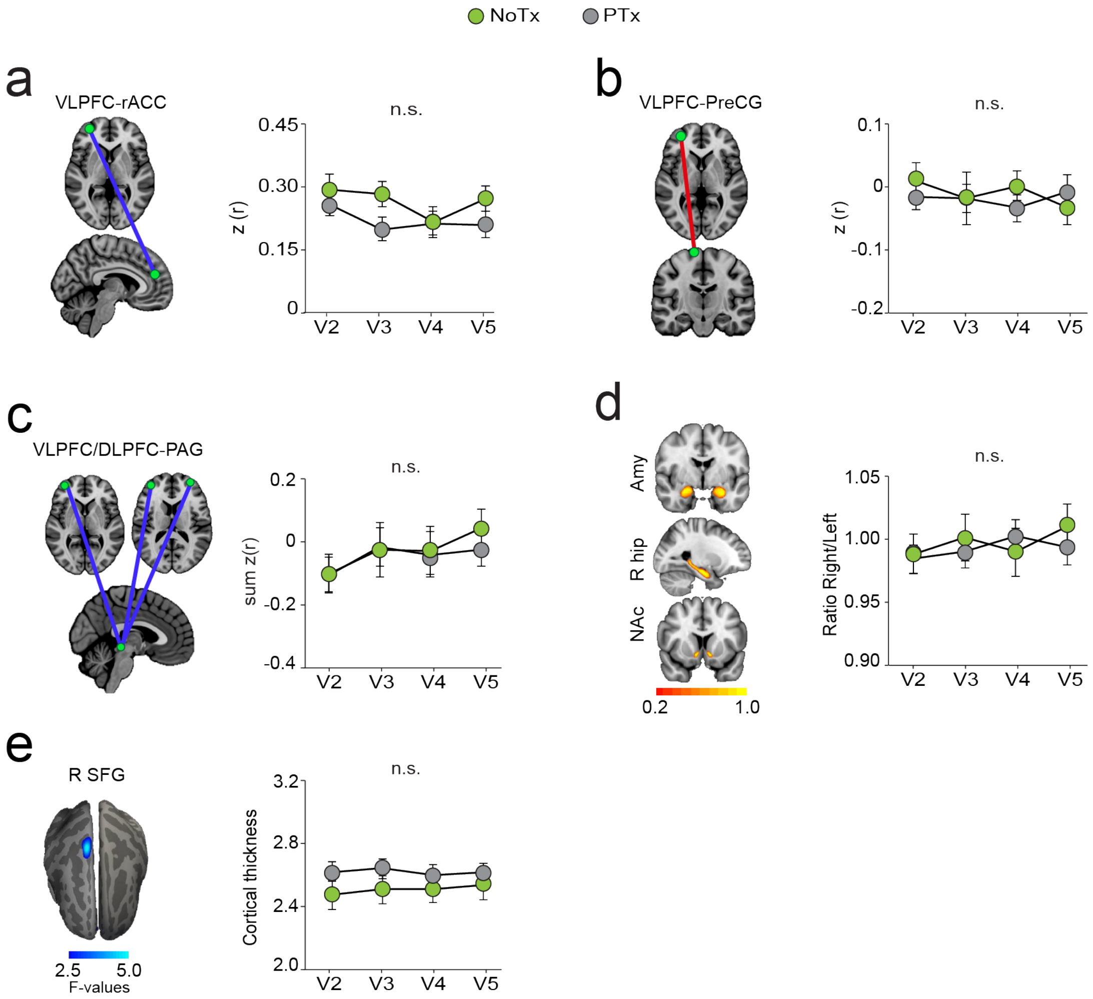

Supplementary Figure 10: Exposure to placebo pills has no causal effects on brain properties determining placebo response. First, the stability of the functional (**a-c**) and anatomical (**d,e**) properties was examined in the NoTx arm (green circles). Repeated-measure ANOVA indicated no effect of time: **a**. VLPFC-rACC ($F_{(2.91, 46.66)} = 1.90$; $p = 0.14$), **b**. VLPFC-PreCG ($F_{(2.39, 33.24)} = 0.44$; $p = 0.66$), **c**. VLPFC/DLPFC-PAg ($F_{(2.70, 45.92)} = 0.60$; $p = 0.60$), **d**. Ratio Right/Left ($F_{(2.38, 42.91)} = 2.93$; $p = 0.06$), **e.** Cortical thickness ($F_{(2.70, 45.92)} = 0.60$; $p = 0.60$). Note that the trending effect of time for the Ratio Right/Left was not supported by the second segmentation software (Freesurfer segmentation Ratio Right/Left: $F_{(2.59, 42.20)} = 0.61$; $p = 0.59$). Second, we tested if exposure to placebo pills had an impact on these brain parameters. The comparison between PTx and NoTx showed no Gr*Time interaction indicating that exposure to placebo pills had no effect: a. VLPFC-rACC ($F_{(2.68, 147.48)} = 1.13$; $p = 0.34$), b. VLPFC-PreCG ($F_{(2.91, 156.92)} = 0.49$; $p = 0.68$), c. VLPFC/DLPFC-PAg ($F_{(2.73, 152.95)} = 0.10$; $p = 0.95$) d. Ratio Right/Left ($F_{(1.98, 107.07)} = 1.60$; $p = 0.21$) e. Cortical thickness ($F_{(2.82, 163.28)} = 0.63$; $p = 0.59$).

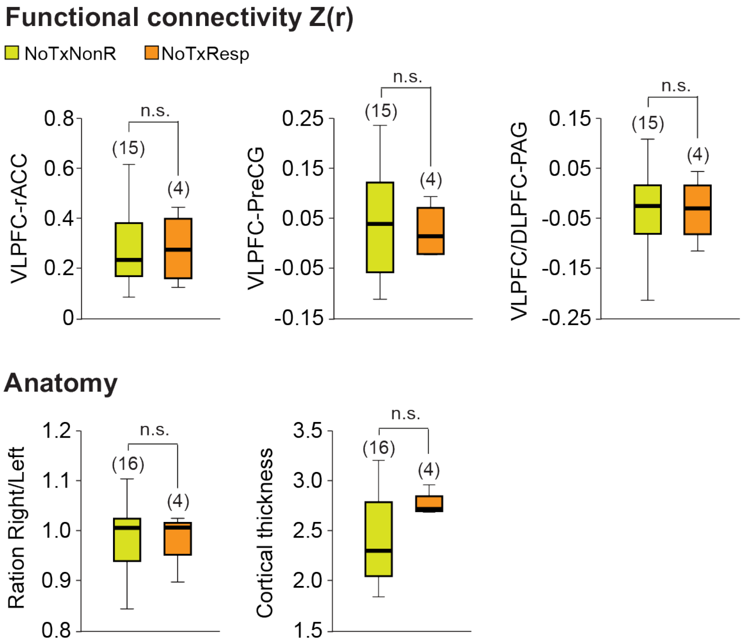

Supplementary Figure 11: Brain properties predicting placebo response were not predicting improvement of symptoms in the no treatment arm. Non-parametric tests revealed that brain parameters had no impact on the response in the NoTx arm: VLPFC-rACC (Mann-Whitney U = 30.00; p = 1.00), VLPFC-PreCG (Mann-Whitney U = 28.00; p = 0.84), VLPFC/DLPFC-PAg (Mann-Whitney U = 37.00; p = 0.81), Ratio Right/Left (Mann-Whitney U = 30.00; p = 0.85), Cortical thickness (Mann-Whitney U = 48.00; p = 0.15).

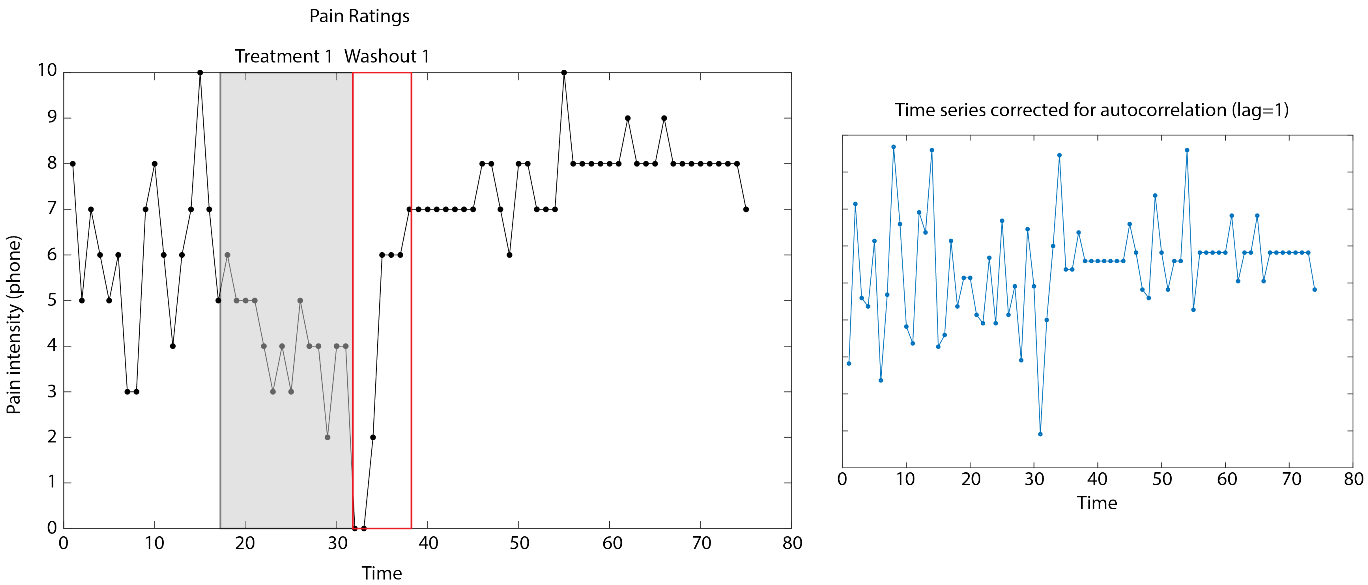

Supplementary Figure 12: Only one subject changed classification after correcting for autocorrelation in the time series of phone app pain ratings. Time series of smartphone app for subject RPL064. The left panel shows the initial pain ratings used to stratify the patients as PTxResp (treatment period shown in grey and washout period squared in red). After correcting for the autocorrelation with a lag of 1 (right panel), the subject no longer showed improvement of symptoms. In that specific case, we visually inspected the data. We did observe high levels of autocorrelation and cycle during the baseline and treatment 1 period. Yet, the pain clearly diminished after introduction of treatment with peak analgesia occurring at the end of the treatment period. More critically, the effect was completely reversed during the washout period. We therefore maintained our initial stratification of RPL064 as a PTxResp. Removing this patient from the analyses did not modify our results.

Data Source	Blind analyses	Follow-up analyses after data un-blinding
**Anatomical data**	- First, we compared the volume of the amygdala, hippocampus, and NAc between the groups. The results showed no significant group differences.<br><br>-Next, we examined hemispheric asymmetry in volumes of amygdala, hippocampus, and NAc.<br><br>-Finally, we compared grey matter density (GMD) within a grey matter mask. Group differences in the correct classifier indicated differences in sensorimotor GMD, but failed to survive correction for multiple comparison.	-We followed up with a vertex-wise whole brain cortical thickness analysis. The analyses were still performed for all 3 classifiers even though the data was un-blinded. Significant results were observed in the sensorimotor community, concordant with the GMD results (**fig. S8**).
**rsfMRI data**	-We performed a permutation test on weighted functional connections between each pair of nodes in pre-determined networks.	
**Questionnaire data**	-Univariate tests for group differences on the scales or subscales as appropriate.	

**Machine learning**		All machine learning models were performed after the data was un-blinded. The performance of group classification was compared to a null distribution generated from scrambled codes.

Supplementary Table 6: Pre-determined plan of analyses. The plan outlined here presents every analysis performed in this study. Everything performed blindly was decided upon prior to study completion. These analyses were performed blindly using 3 classifiers to minimize the effects of bias and expectations. The follow-up analyses were decided after these initial analyses and after data was un-blinded. No analyses were performed outside of the plan. Post-hoc tests were completed after un-blinding the data.

Figure panel	Tests
1c	Independent t-test: treatment 1 $t_{(61)}$ =2.50, p = 0.02 and treatment 2 $t_{(61)}$ =2.95, p = 0.004.
1e	Independent t-test: magnitude of analgesia (left panel) NoTx Vs Tx $t_{(61)}$ =3.30, p = 0.002; PTxNonR Vs PTxResp $t_{(41)}$ =4.77, p < 0.001. Independent t-test: magnitude of %analgesia (right panel) NoTx Vs Tx $t_{(61)}$ =3.60, p < 0.001; PTxNonR Vs PTxResp $t_{(41)}$ =5.38, p < 0.001.
1f	Independent t-test: NoTx Vs Tx $t_{(61)}$ =2.60, p = 0.01; PTxNonR Vs PTxResp $t_{(41)}$ =2.54, p = 0.015.
1g	Independent t-test: treatment 1 NoTx Vs Tx $t_{(57)}$ =1.59, p = 0.12; PTxNonR Vs PTxResp $t_{(38)}$ =1.65, p = 0.12; treatment 2 NoTx Vs Tx $t_{(56)}$ =2.74, p = 0.008; PTxNonR Vs PTxResp $t_{(37)}$ =1.07, p = 0.29.
1h	Independent t-test: treatment 1 NoTx Vs Tx $t_{(57)}$ =0.43, p = 0.67; PTxNonR Vs PTxResp $t_{(38)}$ =3.38, p = 0.002; treatment 2 NoTx Vs Tx $t_{(57)}$ =1.12, p = 0.27; PTxNonR Vs PTxResp $t_{(37)}$ =4.27, p <0.001.
1i	Independent t-test: treatment 1 NoTx Vs Tx $t_{(56)}$ = 1.08, p = 0.29; PTxNonR Vs PTxResp $t_{(38)}$ =0.71, p = 0.49; treatment 2 NoTx Vs Tx $t_{(53)}$ =0.63, p = 0.54; PTxNonR Vs PTxResp $t_{(36)}$ =0.38, p <0.71.
1j	PCA with varimax rotation respectively explained 67% and 65% of the variance of pain outcomes measuring placebo response during treatment 1 and treatment 2. A three-way repeated-measures ANOVA confirmed a significant pain modality (Pain Intensity Vs Pain Quality)*time (6 visits)*Gr interaction ($F_{(10.40, 176.78)}$ = 2.60; p = 0.005) indicating that placebo only impacted pain intensity.
2d	Pearson correlation between magnitude of response with MAIA/e: r = 0.62, ; p < 0.001
2e	Pearson correlation between magnitude of response with MAIA/nd: r = 0.51, p < 0.001.
3a	One-way ANCOVA for subcortical ratio at V2 when controlling for age, sex, and peripheral grey matter (pgrey): $F_{(2,57)}$ = 4.47, p = 0.016; Two-way repeated measure ANCOVA (group after controlling for age, sex, and pgrey): $F_{(1,32)}$ = 6.07, p =0.019.
3b	One-way ANOVA for cortical thickness at V2: $F_{(2,60)}$ = 6.74, p = 0.002; Two-way repeated measure ANOVA (group): $F_{(1,38)}$ = 13.30, p < 0.001.
3c	Correlations between brain anatomy and magnitude of response (Pearson correlation: ratio R/L: r= 0.31; p = 0.049; cortical thickness: r = -0.43; p = 0.004).
4b	Permutation test performed on whole-brain functional connections using the Network Based Statistics toolbox (FDR-corrected q < 0.05).
4d	One-way ANOVA for VLPFC-rACC: $F_{(2,59)}$ = 8.21, p < 0.001; Two-way repeated measure ANOVA (group): $F_{(1,37)}$ = 26.46, p < 0.001.
4e	One-way ANOVA for VLPFC-PreCG: $F_{(2,59)}$ = 4.52, p = 0.015; Two-way repeated measure ANOVA (group): $F_{(1,37)}$ = 9.90, p = 0.003.
4f	One-way ANOVA for VLPFC/DLPFC-PAG: $F_{(2,59)}$ = 7.12, p = 0.002; Two-way repeated measure ANOVA (time*group): $F_{(2.36,89.78)}$ = 2.36, p = 0.09.

4g	Correlations between brain function and magnitude of response (Pearson correlation: DLPFC-rACC: $r= -0.37$; $p = 0.02$; DLPFC-PAG: $r = -0.53$; $p < 0.001$; Spearman: MDLPFC-PreCG: $r = 0.31$; $p = 0.047$ (not shown)).
5b	Z-score 2.68, $p = 0.004$.
5f	Error of the model was assessed with $r^2 = 0.30$; $p < 0.001$
5i	Error of the model was assessed with $r^2 = 0.13$; $p = 0.019$
5k	Error of the combined model was assessed with $r^2 = 0.36$; $p < 0.001$
5l	The difference between predicted response rate in the NoTxResp was not significantly higher than in the NoTxNonR (Fisher exact test $p = 0.29$). Error of the model was assessed with $r^2 =0.01$; $p = 0.53$.

Supplementary Table 7: Details of statistical tests for results shown in figures 1-5.

References

1. Harden RN*, et al.* Medication Quantification Scale Version III: update in medication classes and revised detriment weights by survey of American Pain Society Physicians. *J Pain* **6**, 364-371 (2005).

2. (NCCIH) NCfCaIH. NCCIH Clinical Research Toolbox. (ed^(eds).

3. Melzack R. The short-form McGill Pain Questionnaire. *Pain* **30**, 191-197 (1987).

4. Galer BS, Jensen MP. Development and preliminary validation of a pain measure specific to neuropathic pain: the Neuropathic Pain Scale. *Neurology* **48**, 332-338 (1997).

5. Freynhagen R, Baron R, Gockel U, Tolle TR. painDETECT: a new screening questionnaire to identify neuropathic components in patients with back pain. *Current medical research and opinion* **22**, 1911-1920 (2006).

6. McCracken LM, Vowles KE, Eccleston C. Acceptance of chronic pain: component analysis and a revised assessment method. *Pain* **107**, 159-166 (2004).

7. McCracken LM, Carson JW, Eccleston C, Keefe FJ. Acceptance and change in the context of chronic pain. *Pain* **109**, 4-7 (2004).

8. Jensen MP, Turner JA, Romano JM, Strom SE. The Chronic Pain Coping Inventory: development and preliminary validation. *Pain* **60**, 203-216 (1995).

9. Romano JM, Jensen MP, Turner JA. The Chronic Pain Coping Inventory-42: reliability and validity. *Pain* **104**, 65-73 (2003).

10. Sullivan MJLB, S.R.; Pivik, J. . The Pain Catastrophizing Scale: Development and validation. *Psychological Assessment* **7**, 524-532 (1995).

11. McCracken LM, Dhingra L. A short version of the Pain Anxiety Symptoms Scale (PASS-20): preliminary development and validity. *Pain research & management : the journal of the Canadian Pain Society = journal de la societe canadienne pour le traitement de la douleur* **7**, 45-50 (2002).

12. Ruscheweyh R*, et al.* Validation of the pain sensitivity questionnaire in chronic pain patients. *Pain* **153**, 1210-1218 (2012).

13. Younger J, Gandhi V, Hubbard E, Mackey S. Development of the Stanford Expectations of Treatment Scale (SETS): a tool for measuring patient outcome expectancy in clinical trials. *Clinical trials* **9**, 767-776 (2012).

14. Horne R*, et al.* The perceived sensitivity to medicines (PSM) scale: an evaluation of validity and reliability. *British journal of health psychology* **18**, 18-30 (2013).

15. Hyland ME, Lewith GT, Westoby C. Developing a measure of attitudes: the holistic complementary and alternative medicine questionnaire. *Complementary therapies in medicine* **11**, 33-38 (2003).

16. Wallston KA, Stein MJ, Smith CA. Form C of the MHLC scales: a condition-specific measure of locus of control. *Journal of personality assessment* **63**, 534-553 (1994).

17. Mehling WE, Daubenmier J, Price CJ, Acree M, Bartmess E, Stewart AL. Self-reported interoceptive awareness in primary care patients with past or current low back pain. *Journal of pain research* **6**, 403-418 (2013).

18. Mehling WE, Price C, Daubenmier JJ, Acree M, Bartmess E, Stewart A. The Multidimensional Assessment of Interoceptive Awareness (MAIA). *PloS one* **7**, e48230 (2012).

19. Gross JJ, John OP. Individual differences in two emotion regulation processes: implications for affect, relationships, and well-being. *Journal of personality and social psychology* **85**, 348-362 (2003).

20. Olafsson RP, Smari J, Guethmundsdottir F, Olafsdottir G, Harethardottir HL, Einarsson SM. Self reported attentional control with the Attentional Control Scale: factor structure and relationship with symptoms of anxiety and depression. *Journal of anxiety disorders* **25**, 777-782 (2011).

21. Barry TJH, D.; Lenaert, B.; Debeer, E.; Griffith, J.W. The eACS: Attentional control in the presence of emotion. *Personality and Individual Differences* **55**, 777-782 (2013).

22. Baer RAS, G.T; Hoplins ,J; Krietemeyer, J; Toney, L. . Using Self-Report Assessment to Explore Facets of Mindfulness. *Assessment* **13**, 27-45 (2006).

23. Kotov RIB, S.B.; Watson, D.B. . Multidimensional Iowa Suggestibility Scale (MISS) Brief Manual. (ed^(eds) (2004).

24. Goldberg LR. The development of markers for the Big-Five factor structure. *Psychological Assessment* **4**, 26-42 (1992).

25. Costa PT, & McCrae, R.R. Revised NEO Personality Inventory (NEO-PIR) and NEO Five Factor Inventory (NEO-FFI) professional manual. (ed^(eds). Psychological Assessment Resources (1992).

26. Scheier MFC, Charles S,; Bridges, M. W. . Distinguishing optimism from neuroticism (and trait anxiety, self-mastery, and self-esteem): A reevaluation of the Life Orientation Test. *Journal of personality and social psychology* **67**, 1063-1078 (1994).

27. Berger SE*, et al.* Risky monetary behavior in chronic back pain is associated with altered modular connectivity of the nucleus accumbens. *BMC Res Notes* **7**, 739 (2014).

28. De Baets SB, M. . Development of the loss aversion questionnaire. (ed^(eds School VB) (2012).

29. Beck AT, Steer, R.A. Manual for the Beck Depression Inventory. (ed^(eds). Psychological Corporation (1993).

30. Watson D, Clark LA, Tellegen A. Development and validation of brief measures of positive and negative affect: the PANAS scales. *Journal of personality and social psychology* **54**, 1063-1070 (1988).

31. Power JD*, et al.* Functional network organization of the human brain. *Neuron* **72**, 665-678 (2011).
